# TiO_2_-Based Hybrid Nanocomposites Modified by Phosphonate Molecules as Selective PAH Adsorbents

**DOI:** 10.3390/molecules23113046

**Published:** 2018-11-21

**Authors:** Nadine Bou Orm, Quoc An Trieu, Stephane Daniele

**Affiliations:** 1 College of Natural and Health Sciences, Zayed University, 144534 Abu Dhabi, UAE; Nadine.BouOrm@zu.ac.ae; 2 Institut de Recherches sur la Catalyse et l’Environnement de Lyon (IRCELYON), CNRS—UMR 5256, Université de Lyon, 2 Avenue Albert Einstein, F-69626 Villeurbanne CEDEX, France; quoc-an.trieu@ircelyon.univ-lyon1.fr

**Keywords:** alkoxides, phosphonates, titania, nanohybrid, sol-gel, PAH adsorption, selectivity

## Abstract

A robust sol-gel process was developed for the synthesis of surface-functionalized titania nanocrystallites bearing unsaturated groups starting from molecular heteroleptic single-source precursors. Molecules and nanomaterials were thoroughly characterized by multinuclear liquid and solid-state nuclear magnetic resonance (NMR), infra-red (FT-IR, DRIFT) spectroscopies. Nitrogen adsorption-desorption (BET), thermogravimetric (TG) and elemental analyses demonstrated the reliability and the fine tuning of the surface functionalization in terms of ratio TiO_2_:ligand. The as-prepared materials were used as nano-adsorbents to remove mixture of 16 polycyclic aromatic hydrocarbon (PAHs) from aqueous solutions. Adsorption kinetic experiments were carried out for 24 h in solutions of one PAH [benzo(a)pyrene, 220 ppb] and of a mixture of sixteen ones [220 ppb for each PAH]. Most kinetic data best fitted the pseudo-second order model. However, in PAHs mixture, a competition process took place during the first hours leading to a remarkable high selectivity between light and heavy PAHs. This selectivity could be fine-tuned depending on the nature of the unsaturated group of the phosphonate framework and on the nanomaterial textures.

## 1. Introduction

Polycyclic aromatic hydrocarbons (PAHs) are a family of nonpolar neutral chemical compounds resulting from the condensation of several benzene rings ranging from two to three (light molecular weight PAHs, LMW PAHs), to four and five (medium molecular weight PAHs, MMW PAHs) and up to seven (heavy molecular weight PAHs, HMW PAHs). The presence of PAHs in surface water comes mainly from the deposition of airborne particles produced via incomplete oil combustion, from the leaching releases from coal storage areas, from effluents of wood treatment plants and other industries and from the use of composts and fertilizers [1] The number of PAHs identified is in the order of 130, including PAHs containing sulfur, nitrogen or oxygen atoms. The U.S. Environmental Protection Agency (US-EPA) has compiled a list with 16 PAHs (Table S1) posing major environmental problems due to their persistence, their toxic effects on human health and are associated with a wide range of effects: degradation of the immune system, effects on the reproduction and development as endocrine disrupter and carcinogenic properties (carcinogenicity increasing for HMW PAH) [2].

PAHs are persistent pollutants in the environment since they are often resistant to biodegradation. Their removal using conventional physical and/or chemical technologies available for remediation of wastewater pollutants such as oxidation with ozone/hydrogen peroxide, photocatalytic degradation, ion exchange, membrane (ultra)filtration, coagulation/flocculation, solvent extraction and reverse osmosis can also be not efficient. However, solid-phase extraction is generally considered to be superior for water re-use in terms of energy costs, flexibility and simplicity of design, ease of operation and insensitivity to toxic pollutants and because it does not result in the formation of harmful by-product or toxic concentrated sludge [3,4,5].

Hybrid (organic-inorganic) nanostructured materials of class II combining large surface-to-volume ratio and controllable surface functionalization can provide a powerful potential to update traditional adsorption treatment process. One of the goals of nanotechnology for the pollutant removal from wastewater is to develop efficient and selective nanosorbents that can remove low concentration contaminants in the presence of competing ones. A recent study on the removal selectivity of PAHs from a wastewater treatment plant (WWTP) located in an urban area in China shows that LMW PAHs such as naphthalene can be removed at a level of 80%, while HMW ones became even more concentrated with treatment in both the dissolved phase and the dewatered sludge [6]. Given the nature of PAHs, use of supported-aromatic derivatives to form "π-stacking" charge transfer complexes has already been reported since these interactions are effective in an aqueous medium, non-degrading and reversible [7,8,9]. Previous studies have reported the removal of PAHs from aqueous solutions using a large variety of supports such as clay, resins, activated carbon, periodic modified mesoporous organosilane (POMs), metal-organic frameworks (MOFs) [4,5,10]. However, studies dealing with the influence of the nature of the trapping organic molecule on adsorption properties towards PAH selectivity or affinity in mixture of LMW and HMW ones are scarce.

In this study, we report the synthesis, characterization and properties of new hybrid (in this case organic-inorganic) nanotitania supports functionalized with a series of organophosphorus molecules (R–OPO_2_H_2_) holding different unsaturated groups (R = vinyl, phenyl, naphtyl) and spacers. The performances of such π-stacking complexation sites will be compared in terms of selectivity, capacity and kinetic of adsorption in an aqueous mixture of 16 PAH molecules. There is an abundant literature reporting the use of organophosphorus molecules for surface modification of metal oxides (MO_x_) because they allow the control of a mono-layer functionalization of metal oxide surfaces resulting from the non-competition between hetero-and homo-condensation but also but also because this ionocovalent anchoring is highly stable through multiple M-O-P bonds [11,12]. In particular, the Titanium oxide will be our nano-support of choice, because of the simple "soft chemistry" preparation routes of crystallized nanoparticles of titanium oxide widely studied in the literature and in our laboratory [13].

## 2. Results and Discussion

### 2.1. Heteroleptic Precursors Synthesis

A series of heteroleptic phosphonate derivatives of general formulae [Ti_2_(OiPr)_6_(O_3_P-R)]_m_ [R = C_2_H_3_ (**1**), C_6_H_5_ (**2**), CH_2_CH_2_NHCH_2_(C_10_H_7_) (**3**), CH_2_CH_2_(NC_9_H_10_) (**4)**] was synthetized by Brönsted acid-base reactions between Ti(OiPr)_4_ and 0.5 equivalents of the corresponding phosphonic acid (Table 1), at ambient temperature in isopropanol and under an argon atmosphere (Equation (1)).

(1) $$2m\;\mathrm{Ti}{\left(\mathrm{OiPr}\right)}_{4}+m\;{\left(\mathrm{HO}\right)}_{2}\;\mathrm{OP}-R\overset{i\mathrm{Pr}\mathrm{OH}}{\underset{20{}^{\circ}C}{\to }}{\left[{\mathrm{Ti}}_{2}{\left(\mathrm{OiPr}\right)}_{6}\left(O_{3}P-R\right)\right]}_{m}+2m\;\mathrm{HOiPr}$$

R = C_2_H_3_ (1); R = C_6_H_5_ (2); R = CH_2_CH_2_NHCH_2_(C_10_H_7_) (3); R = CH_2_CH_2_(NC_9_H_10_) (4)

Higher equivalents of phosphonic acid led to insoluble precipitates. After a drying process, all products were orange-brown liquids. Compounds **1** and **2** were soluble in THF while **3** and **4** were only soluble in hot isopropanol.

All the derivatives were obtained in quantitative yields without purification step and were fully characterized by FT-IR, ^1^H- and ^31^ P-NMR and P, Ti elemental analyses. All FT-IR spectra exhibited absorption bands at 1260 cm^−1^, between 1200 and 950 cm^−1^ and below 800 cm^−1^ corresponding to P=O, P–O and Ti–O bonds, respectively. The ^1^H-NMR spectrum of **1**, at room temperature in CDCl_3_, displayed a 36:6:3 relative integration between CH_3_, CH and vinylic, respectively, in agreement with the proposed general formulae [Ti_2_(OiPr)_6_(O_3_PC_2_H_3_)]_m_. The ^31^P-NMR spectrum showed the presence of only a single signal at 7.46 ppm, high fielded in comparison to the vinyl phosphonic acid at 18.88 ppm. Same characteristics were observed for all the derivatives **2**–**4** and were in agreement with the literature data [11].

### 2.2. Hybrid Nanomaterials Synthesis

Derivatives **1**–**4** have been used as hydrolysable precursors in a further sol-gel step to afford hybrid (TiO_2_)_x_(O_3_P-R) nanomaterials, following a procedure we have already described for titania carboxylic surface modification [14] (Equation 2). By varying the co-hydrolysis step parameters, we have checked the influence of the amount and/or the nature of R moiety towards the performance of the resulting nanomaterials as nano-absorbents for a mixture of 16 PAHs.

(2) $${\left[{\mathrm{Ti}}_{2}{\left(\mathrm{OiPr}\right)}_{6}\left(O_{3}P-R\right)\right]}_{m}+y\;\mathrm{Ti}{\left(\mathrm{OiPr}\right)}_{4}\overset{1)i\mathrm{Pr}\mathrm{OH}}{\underset{2)H2O,100{}^{\circ}C\;NR\;4Br}{\to }}{\left(\mathrm{Ti}O_{2}\right)}_{X}\left(O_{3}P-R\right)$$

#### 2.2.1. Syntheses and Characterization of (TiO_2_)_x_(VPA) Samples (Ti/P ratio (x) = 2–200)

The first parameter to modify was the amount of vinyl phosphonic acid (VPA) by co-hydrolyzing the derivative **1** with different amounts of Ti(OiPr)_4_ in order to produce white powders with controlled Ti/P ratio (x) from 2 to 200. All X-ray powder diffractograms corresponded to the anatase phase of TiO_2_ (file ICDD PDF-4+, 00-21-1272) with crystallite sizes estimated by the Debye-Scherrer equation at around 4–6 nm in size (Figure S1). This result suggested that the crystallite size of TiO_2_, the nature of the phase and the crystallinity were not affected by the presence of the phosphonic acid graft up to a certain limit, since the highest concentrated phosphonic acid sample (x = 2) was amorphous. Unlike pure TiO_2_, no trace of the brookite phase was detected.

**Figure 1:**
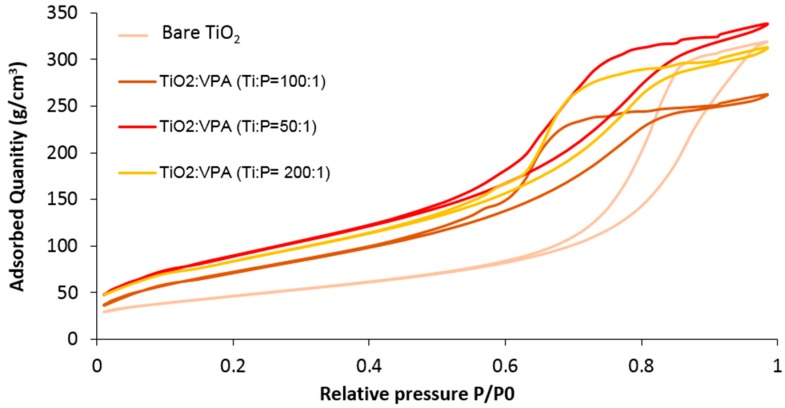
N_2_ adsorption-desorption isotherms for bareTiO_2_, and hybrid samples (TiO_2_)_x_(VPA) with x = 50, 100 and 200.

Figure 1 shows the nitrogen adsorption-desorption isotherms for the bare TiO_2_ and some samples (TiO_2_)_x_(VPA) (x = 50, 100, 200) desorbed at 100 °C for 6 h. All displayed type IV(a) isotherms with an H2(b)-type hysteresis loop (according to IUPAC terminology) [15], typical for mesoporous materials with complex pore-networks. Data showed a slight increase of the specific surface area up to 269–333 m^2^/g for hybrid samples and that these latter exhibited larger final saturation plateau than bare TiO_2_, suggesting higher inter-particle interactions and extra-mesoporosity.

The most important study remained the surface characterizations in order to compare the different sample natures and to correlate them to the synthesis procedure and to their final properties as PAH nano-absorbents.

**Figure 2:**
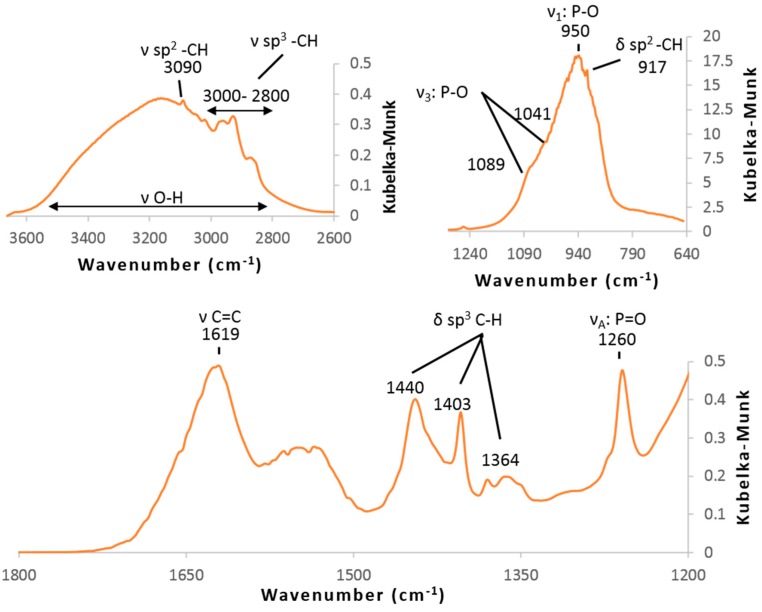
DRIFT spectra of samples (TiO_2_)_100_(VPA) at 150 °C.

All FT-IR spectra exhibited strong absorption bands ascribed to Ti-O and O-H bonds below 800 cm^−1^, around 1620 cm^−1^ and above 2800 cm^−1^, (Figure S2), however, the concentrations of VPA were too small to display clear absorption bands assigned to O–P–O group between 1200 and 950 cm^−1^. Only a weak band at 1260 cm^−1^ was related to P=O bond. Phosphonate and C=C bonds were evidenced by DRIFT experiments under argon at 150 °C since spectra displayed strong adsorption bands at around 1000 cm^−1^ (symmetrical and unsymmetrical stretching band of O–P–O) and weak ones at 1260 cm^−1^ (νP=O), and 1619 cm^−1^ (νC=C). (Figure 2) Absorption bands in the range of 3000–2800 cm^−1^ (C-H bending) and 1500–1350 cm^−1^ (C-H stretching) could be explained by the presence of residual alcohol molecules at the surface.

In an attempt to quantify the final VPA content or at least to estimate its control, TGA-TDA experiments of the (TiO_2_)_x_(VPA) samples were performed under air from 20 to 600 °C. All the patterns were similar and displayed two major weight losses ranging from 20 to 170 °C (endothermic step) and from 170 to 500 °C (exothermic step), corresponding to the removal of water and solvents (alcohols) adsorbed and to the pyrolysis the hydroxyl groups (–OH) and surface vinyl phosphonates, respectively (Figure S3).The relationship between the organic content and the weight loss observed in TGA was difficult to establish since phosphate ligands (PO_4_) still remained onto the TiO_2_ surface until 900 °C.

**Figure 3:**
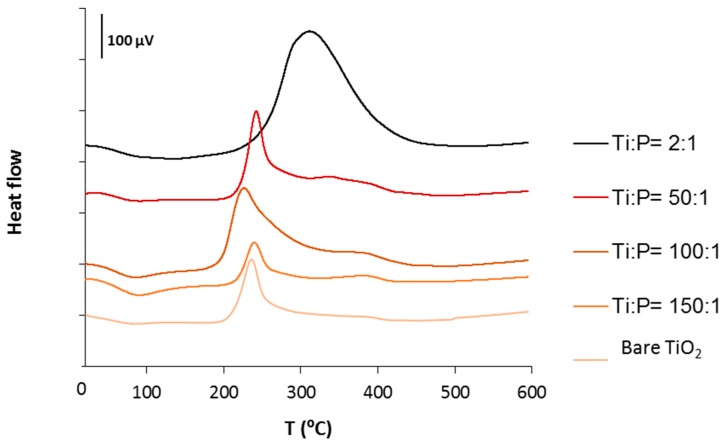
TDA of bare and some hybrid TiO_2_ samples (samples Ti/P = 25; 75, 125 and 200 are omitted for clarity).

However, in comparison with the TDA patterns of the richest VPA sample (from the hydrolysis of **1**) and the bare TiO_2_, one could estimate that the removal of vinyl phosphonic organic units occurred mainly between 300 and 450 °C. The increase in the organic content at the surface was also accompanied by a reduction in the intensity of the TDA peak between 200 and 275 °C, associated to the loss of –OH groups (Figure 3).

**Figure 4:**
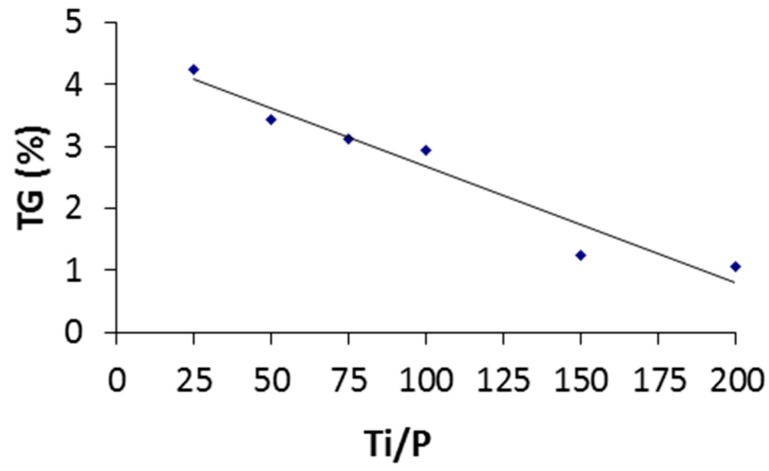
Variation of organic weight loss in the range of 300–500 °C in thermogravimetric analysis as a function of the starting Ti/P ratio.

Then, by plotting the weight loss between 300 and 500 °C versus the starting Ti /P ratios (Figure 4), one could demonstrate that weight losses followed a linear trend (y = −0.0189x + 4.5638) with a high regression coefficient R^2^ = 0.94.

The most loaded sample (Ti/P = 25) having the highest weight loss of 4.25%, while for higher Ti/P ratios, a phenomenon of dilution of the phosphonate ligands at the TiO_2_ surface logically decreased the percentages down to 1.05%. Such thermal analysis has thus demonstrated that the phosphonate loading in the hybrid nanomaterial has varied constantly with the initial stoichiometry of the solutions of hydrolysis (Ti/P ratio) and therefore, the degree of surface functionalization of TiO_2_ nanocrystallites could be controlled, to some extent, by the initial concentration of the heteroleptic precursor holding the phosphonate moiety. Ti and P elemental analyses on the samples (TiO_2_)_x_(VPA) were carried out and exhibited close values to the expected ones (Table 2).

^31^P {^1^H} MAS NMR experiments exhibited similar patterns show two distinct resonances upfield and downfield shifts (lower and higher ppm) at about 19 and 13 ppm and broadened compared to the resonance of the original ligand VPA at about 18 ppm (Figure S4). These data, in agreement with the data given in the literature, did again confirm the phosphonate grafting onto the TiO_2_ surface nanoparticles [16,17]. The width of the peaks and the presence of P=O absorption band in DRIFT spectra (Figure 2) suggested a mixture of bidentate and tridentate surface grafting modes [11]. The data also demonstrated the absence of molecular titanium phosphonate phases formed by dissolution-precipitation process which are expected to give peak at higher field such as layered titanium phenyl-phosphonates at −4.0 ppm [18].

In an attempt to choose an appropriate Ti/P ratio for the following adsorption study, we estimated the density of VPA molecules onto 5 nm TiO_2_ nanocrystallite surface. The intermediate Ti/P ratio of 100 was selected for comparison because (i) sample contained enough phosphonic acid ligand to show an effect and (ii) only 12% of Ti atoms were coordinated or 5 % of the surface were covered, assuming only tridentate bonding mode or an area of 24 Å^2^ per molecule of phosphonic acid, respectively. This would achieve high dispersion of the un-saturated receptor, avoiding any side π−π stacking effect between aromatic rings onto the surface. In the same way, Stellaci et al. have demonstrated the use of mixed-ligand gold-coated nanoparticles (short and long thioethers) as efficient receptor for PAH because of supramolecular effect [19]. So for the following syntheses, the theoretical ratio Ti/P was kept constant to 100.

#### 2.2.2. Syntheses and Characterizations of (TiO_2_)_100_(O_3_P-R) Samples

Following the same procedure as for the VPA ligand, a series of three new hybrid nanomaterials of general formulae (TiO_2_)_100_(O_3_P-R) was synthetized as white powders. The elemental analyses data confirmed a Ti/P ratio close to 100 (Table 3).

Powder XRD (Figure S5), FT-IR (Figure S6), TGA-TDA (Figure S7) and solid state ^31^P {^1^H} MAS NMR (Figure S8) data were consistent with the results already obtained with the VPA ligand. In addition, nanomaterials belonging aromatic rings such as (TiO_2_)_100_(PPA), (TiO_2_)_100_(NMAPA), and (TiO_2_)_100_(HQPA), displayed a characteristic shift of the carbons linked to the aromatic ring at δ = 128 ppm in the solid state ^13^C {^1^H} MAS NMR spectra (Figure S9).

**Figure 5:**
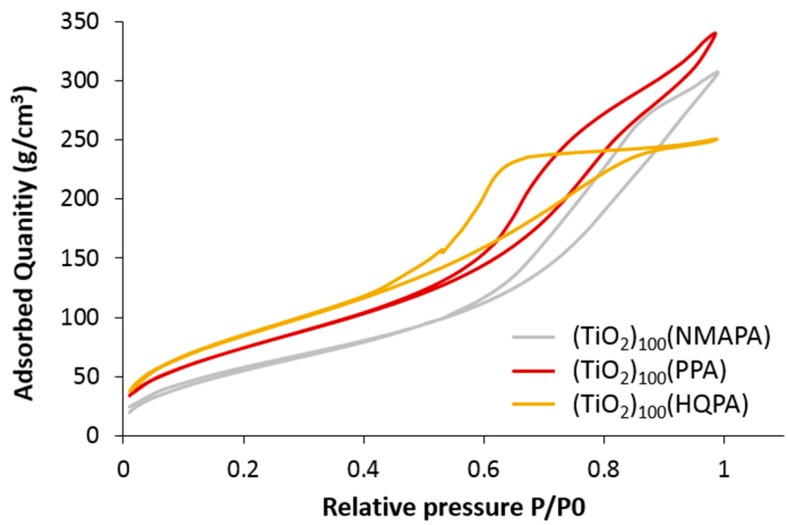
Nitrogen adsorption-desorption isotherms of (TiO_2_)_100_(PPA), (TiO_2_)_100_(NMAPA), (TiO_2_)_100_(HQPA).

Similar to VPA–based hybrid nanomaterials, the three new hybrid nanomaterials exhibited close specific surface area (225–322 m^2^/g) and volume pore (0.39–0.51 cm^3^/g) and similar type IV(a) isotherms than bare TiO_2_ (Figure 5 and Table S2). However, (TiO_2_)_100_(PPA) and (TiO_2_)_100_(NMAPA) materials displayed different adsorbent textures with type H3 hysteresis loops suggesting the presence of macroporosity [20].

### 2.3. Kinetic PAH Adsorption Studies of (TiO_2_)_100_(O_3_P-R) Samples

**Figure 6:**
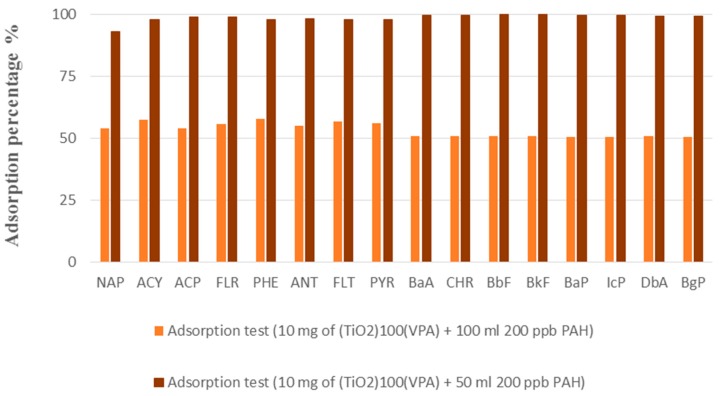
Batch adsorption tests of (TiO_2_)_100_(VPA) after 24 h.

Batch absorption tests using 10 mg of (TiO_2_)_100_(VPA) and 50 mL of 200 ppb PAH aqueous solution for 24 h at 20 °C and at pH 6.5, showed that the 16 PAH molecules were extracted with efficiencies of 93–100% leading to a high total equilibrium adsorption capacity of around 16–17 mg·g^−1^ (or around 1 mg·g^−1^ of each PAH). This was confirmed by doubling the volume of PAH aqueous solution, using 100 mL batches of 200 ppb PAH solution with 10 mg of the materials, whereupon the adsorption percentage was half that of the previous tests (Figure 6).

Close values were reported for nano-hybrids NH_2_-SBA-15 towards three PAHs (naphthalene, acenaphthylene and phenanthrene) [22] and periodic mesoporous organosilica functionalized with phenyl groups towards a mixture of five PAHs (naphthalene, acenaphthene, fluorine, fluoranthene, pyrene) [23] with adsorption capacity ranges of 0.76–1.92 mg g^−1^ and 0.72–1.69 mg g^−1^, respectively. Nano-absorbents combining both –NH_2_ and phenyl groups such as hybrid mesoporous *p*-aminobenzoic acid-MCM-41 (PABA-MCM-41) nano-materials were used to remove benzo[k]fluoranthene and benzo[b]fluoranthene from aqueous media [200 ppb] and have exhibited lower adsorption capacity in the range of 23–27 μg g^−1^ [24].

Adsorption kinetics studies were then carried out to determine the time required to reach adsorption equilibrium, the extraction efficiencies and the selectivities towards the PAHs of the different hybrid sorption systems.

**Figure 7:**
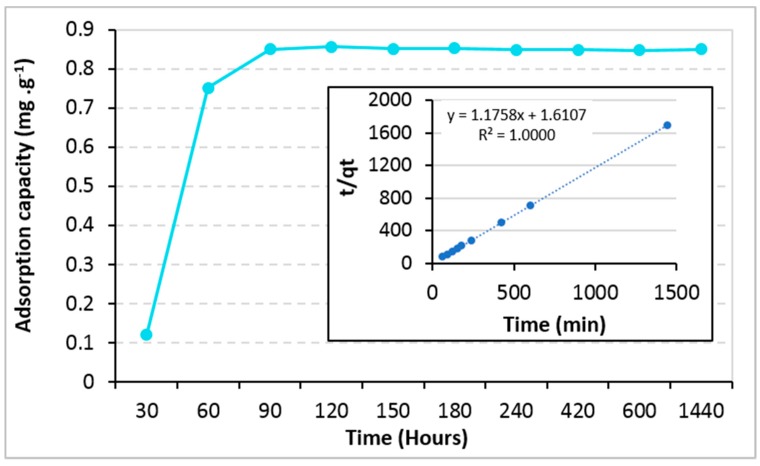
Adsorption capacity of (TiO_2_)_100_(VPA) toward benzo(a)pyrene as a function of time. Insert: Pseudo-second model for benzo(a)pyrene adsorption kinetics.

We carried out the experiment with (TiO_2_)_100_(VPA) in an aqueous solution containing only one PAH [i.e. benzo(a)pyrene] at a concentration of 220 ppb. The pattern showed that the equilibrium was reached after 90 min leading to an equilibrium capacity of 0.97 mg·g^−1^. Figure 7 showed that experimental data fitted very well with the pseudo-second order model (R^2^ value of 1.000) (Table S3) as largely reported for other absorbents such as zeolite, clays, sepiolite, activated carbon, biosorbents and hybrid mesoporous material [5]. Thanks to high surface area of (TiO_2_)_100_(VPA) nano-materials and stirring during the adsorption, mass transfer process of adsorbates to nano-TiO_2_’s surface was negligible that left interaction between adsorbates and active site on surface of adsorbent and adsorbate as a rate-limiting step in the adsorption process.

**Figure 8:**
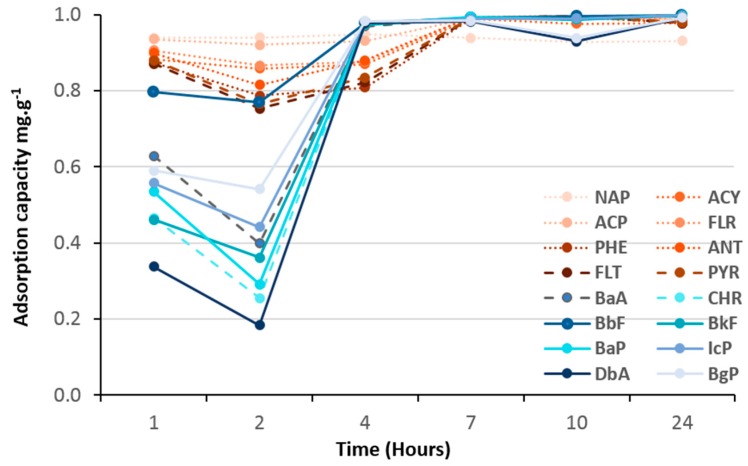
Adsorption kinetics of 16 PAHs on (TiO_2_)_100_(VPA).

**Figure 9:**
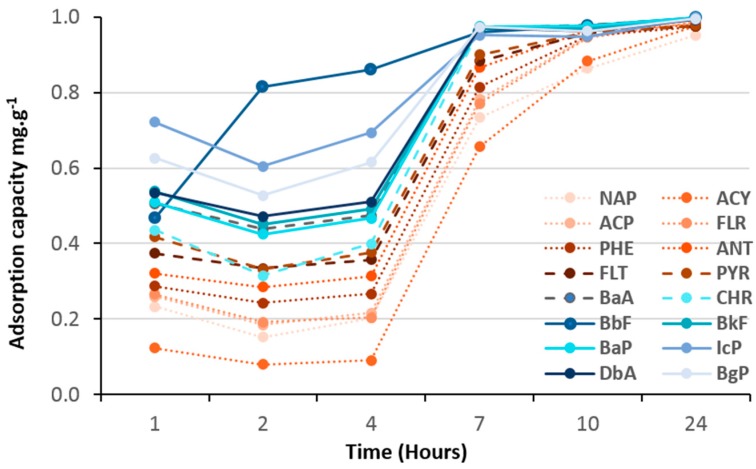
Adsorption kinetics of 16 PAHs on (TiO_2_)_100_(PPA).

**Figure 10:**
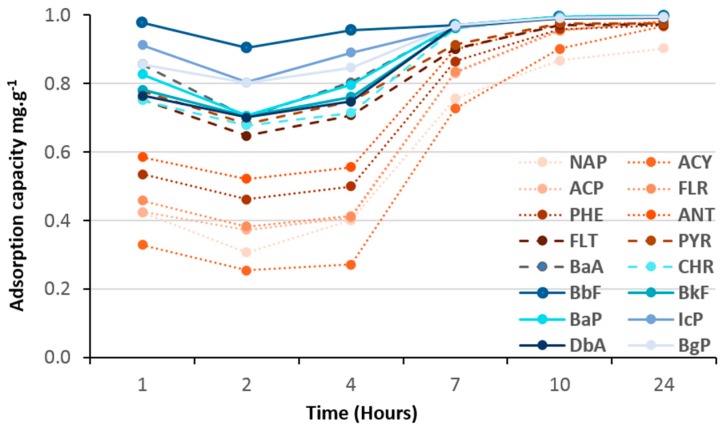
Adsorption kinetics of 16 PAHs on (TiO_2_)_100_(NMAPA).

**Figure 11:**
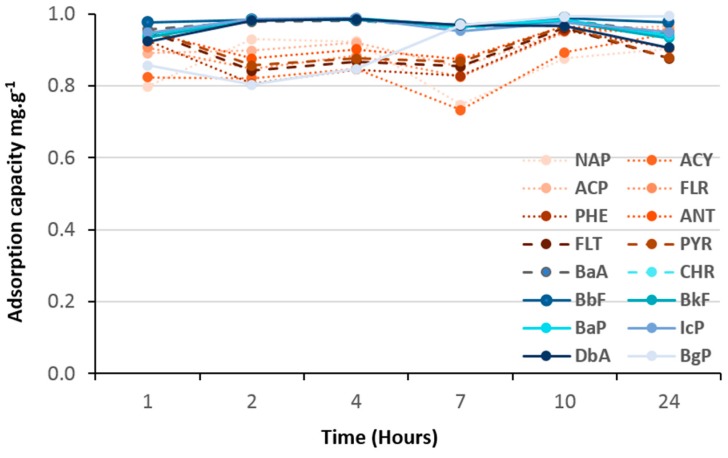
Adsorption kinetics of 16 PAHs on (TiO_2_)_100_(HQPA).

As shown in Figure 8, Figure 9, Figure 10 and Figure 11, all hybrid (TiO_2_)_100_(O_3_P-R) nanomaterials demonstrated remarkable 100% of removal efficiency of all 16 PAHs after 24 h. Such results confirmed that an aromatic function was not necessary to have efficient charge transfer, as reported by Bloom et al [9].

Adsorption kinetics of VPA-, PPA- and NMAPA-based material followed similar and two-stage patterns with first one ascribed as a competition between light, medium and heavy PAHs followed by an equilibrium one to reach 100% of adsorption of all PAHs. Interestingly, while VPA-based (vinyl) nanomaterial had more affinity with light PAH molecules in the first seven hours, going through PPA (phenyl) to NMAPA (naphtyl) gave totally opposite adsorption kinetic behaviors. In particular the NMAPA-based ligand displayed the highest affinity for the heavy PAHs during the first hours of experiment. We could ascribe this result to strongest quadrupole interactions between naphtyl groups (two cycles) and PAHs with several aromatic cycles than vinyl ones. It was also well-known that the most stable conformations of benzene dimers are parallel displaced or T-shaped compared to sandwich one [24]. One could speculate that these conformations led to different steric hindrance at the surface of the nano-materials and time was mandatory to reach different supramolecular assembly organizations. Other parameter that could be taken into account was the change of the adsorbent texture since PPA-and NMAPA-based mesoporous materials exhibited type H3 hysteresis loops consisting in the presence of macroporosity (more convenient for the diffusion of heavy PAHs) while VPA one exhibited type H2(b) hysteresis loop revealing small pore neck diameters (cavitation and pore blocking effects) [20]. We have already reported how supramolecular spatial arrangement of hybrid titania nanocrystals through the use of bi-functional ligands (DTPA) enabled to reach efficient lanthanide ionic separation with very high selectivity (i.e., S_La/Gd_ = 160) [25].

Finally, (TiO_2_)_100_(HQPA) sample has shown more affinity to heavy PAH but did not exhibit any competition process during the first hours, adsorption percentages of all PAH being constant all over the experiment (Figure 11). In comparison with the PPA-based sample containing also one phenyl ring, this last result demonstrated that subtle change of the phenyl 3D spatial organization and/or texture [from H3 to H2(b) hysteresis loop] might drastically impact the selectivity of our hybrid nano-absorbents towards mixture of PAHs.

Thermodynamic studies and computational calculations are now under progress in order to elucidate whether mono or multilayer or T-shaped or parallel displaced conformations could be at the origin of such selectivity performances.

## 3. Experimental Section

### 3.1. General Information

All manipulations have been carried out under an argon atmosphere using standard Schlenk and glovebox techniques. THF and HOiPr were dried using either an MBRAUN SPS-800 system (Garching, Germany) (THF) or distillation over [Al(OiPr)_3_]_4_, respectively. Deuterated solvents (CDCl_3_, MeOD) were stored over molecular sieves. Vinyl phosphonic acid (VPA), phenyl phosphonic acid (PPA) and ammonium salt (N*n*Bu_4_Br) were purchased from Sigma Aldrich (Saint-Louis, MO, USA) and were used as received. Phosphonic acids such as (C_10_H_7_)CH_2_NHCH_2_CH_2_PO(OH)_2_ (NMAPA), (C_9_H_10_N)CH_2_CH_2_PO(OH)_2_ (HQPA) were synthesized according to the published procedure and stored under argon [26]. Ti(OiPr)_4_ (TTIP) was purchased from Sigma Aldrich and distilled under vacuum prior used. The standard stock solution containing sixteen US-EPA PAHs at a concentration of 500 ppm each was purchased from Sigma Aldrich. It was diluted to a concentration of 200 ppb (μg L^−1^) each in deionized water: ethanol mixture prior used in extraction tests.

### 3.2. Product Characterizations

^1^H liquid and ^13^C solid state MAS NMR spectra were recorded on an AM-250 spectrometer Bruker (Billerica, MA, USA). ^31^P solid state MAS NMR were recorded on a Bruker DSX400 spectrometer spectra with a 4 mm probe and rotation speeds of 5 to 10 Kz. Analytical data were obtained from the Institut des Sciences Analytique (UMR 5280, Villeurbanne, France) by ICP. FT-IR spectra were recorded as Nujol suspensions (for air sensitive derivatives) or as KBr pellets (for hydrolyzed powders) on a Bruker Vector 22 FT-IR spectrometer at room temperature and registered from 4000 to 400 cm^−1^. DRIFT experiments were carried out on a Nicolet 6700 FTIR spectrophotometer (Thermo Fischer, Waltham, MA, USA) equipped with a MCT detector (number of scan: 64, resolution: 4 cm^−1^). The recording mode was diffuse reflectance and the optical unit, Praying Mantis (Harrick). TGA/TDA data were collected with a model 92 system (Setaram, Lyon, France) in air with a thermal ramp of 10 °C min^−1^. Powder X-ray diffraction data were obtained with a D5005 diffractometer (Siemens, Munich, Germany) by using Cu-*K*α radiation at the scan rate of 0.02°2*θ* s^−1^ with step times of 1 and 16 s for routine or for expanded region spectra, respectively. BET experiments were carried out with an ASAP 2010 system after the samples were desorbed at 100 °C for 6 h.

### 3.3. Synthesis of Titanium Precursors

#### 3.3.1. Synthesis of [Ti_2_(OiPr)_6_(O_3_PC_2_H_3_)]_m_ (**1**)

Under an argon atmosphere Ti(OiPr)_4_ (1.48 mL, 4.9 mmol) was dissolved in anhydrous THF (10 mL). In another Schlenck flask, CH_2_=CH-PO(OH)_2_ (272 mg, 2.5 mmol) was added to anhydrous THF (23 mL). Under an inert atmosphere, the vinyl phosphonic acid solution was added dropwise to the Ti(OiPr)_4_ one. The mixture was stirred at room temperature for 18 h. After evaporation of the solvent, 1.62 g (quantitative yield relative to titanium) of **1**, soluble in THF, were obtained as a brown oil. Anal. Calcd for C_20_H_45_O_9_PTi_2_: Ti: 17.20; P: 5.56; Found Ti: 17.24, P: 5.50. ^1^H-NMR (20 °C, CDCl_3_); δ ppm 6.25–5.70 (m, 3H, –CH=CH_2_); 5.05–4.30 (m, 6H, –OCH); 1.45–1.06 (m, 36H, –CH_3_). ^31^P {^1^H}-NMR (20 °C, CDCl_3_); δ ppm: 7.46. FT-IR (Nujol), cm^−1^: 510 (s), 616 (s), 710 (m), 748 (m), 797(s), 851 (m) (ν Ti–O); 955 (s), 1008 (vs), 1056 (s), 1082 (s) (ν P–O); 1126 (vs), 1151 (sh), 1179 (sh) (ν C–O); 1261 (m) (ν P=O); 1331 (w); 1615(w) (ν C=C).

#### 3.3.2. Synthesis of [Ti_2_(OiPr)_6_(O_3_PC_6_H_5_)]_m_ (**2**)

Using the same procedure as previously described but with Ti(OiPr)_4_ (1.51 mL, 5 mmol) dissolved in anhydrous THF (6 mL) and C_6_H_5_PO(OH)_2_ (395 mg, 2.5 mmol) in anhydrous THF (17 mL) gave 1.76 g (quantitative yield relative to titanium) of **2** as a light brown oil soluble in THF. Anal. Calcd for C_24_H_47_O_9_PTi_2_ Ti: 15.79, P: 5.11; Found Ti: 15.82, P: 5.13. ^1^H-NMR (20 °C, CDCl_3_); δ ppm: 7.90–6.80 (m, 5H, –C_6_H_5_); 5.14–4.23 (m, 6H, –OCH); 1.45–1.06 (m, 36H, –CH_3_). ^31^P {^1^H}-NMR (20 °C, CDCl_3_); δ ppm: 9.70. FT-IR (Nujol), cm^−1^: 612(m), 625 (m), 702 (m), 748 (m), 838 (m), 853 (m) (ν Ti–O); 957 (s), 976 (s), 1009 (vs), 1034 (m), 1089 (s) (ν P–O); 1135 (vs), 1162 (sh) (ν C–O); 1257 (m) (ν P=O); 1320 (w); 1630 (w) (ν C=C).

#### 3.3.3. Synthesis of [Ti_2_(OiPr)_6_(O_3_PCH_2_CH_2_NHCH_2_(C_10_H_7_)]_m_ (**3**)

Using the same procedure as previously described with (C_10_H_7_)CH_2_NHCH_2_CH_2-_PO(OH)_2_ (0.290 g, 1 mmol) dissolved in hot isopropanol (20 mL) and Ti(OiPr)_4_ (0.268 g, 2 mmol) in isopropanol (3 mL) under reflux for 8 h gave 0.83 g (quantitative yield relative to titanium) of **3** as an orange oil soluble in isopropanol under reflux and insoluble in THF. Anal. Calcd for C_31_H_56_NO_9_PTi_2_Ti: 12.94, P: 4.18; Found Ti: 12.97, P: 4.14. ^1^H-NMR (20 °C, MeOD); δ ppm: 8.12 (m, 1H, –C_10_H_7_); 7.97–7.12 (m, 6H, –C_10_H_7_); 4.67–4.34 (m, 2H, –NC*H*_2_); 4.16–3.70 (m, 6H, –OCH); 3.42 (d, *J* = 33.5 Hz, 2H, –C*H*_2_); 2.04 (dd, *J* = 35.3; 28.3 Hz, 2H, –PCH_2_); 1.38–0.64 (m, 36H, –CH_3_). ^31^P {^1^H}-NMR (20 °C, MeOD); δ ppm: 7.57. FT-IR (Nujol), cm^−1^: 614(m), 624 (m), 708 (m), 748 (m), 843 (m), 853 (m) (ν Ti–O); 956 (s), 977 (s), 1008 (vs), 1045 (m), 1086 (s) (ν P–O); 1125 (vs), 1162 (sh) (ν C–O); 1256 (m) (ν P=O); 1325 (w); 1631 (w) (ν C=C).

#### 3.3.4. Synthesis of [Ti_2_(OiPr)_6_(O_3_PCH_2_CH_2_(NC_9_H_10_)]_m_ (**4**)

Using the same procedure as previously described with (C_9_H_10_N)CH_2_CH_2_PO(OH)_2_ (0.256 g, 1 mmol) n hot isopropanol (20 mL) and Ti(OiPr)_4_ (0.268 g, 2 mmol) in isopropanol (3 mL) under reflux for 8 h gave 0.80 g (quantitative yield relative to titanium) of **4** as an orange oil soluble in isopropanol under reflux and not soluble in THF. Anal. Calcd for C_29_H_56_NO_9_PTi_2_ Ti: 13.84, P: 4.48; Found Ti: 13.78, P: 4.50. ^1^H-NMR (20 °C, MeOD); δ ppm: 7.68–7.42 (m, 2H, C_6_H_4_); 7.08 (dt, *J* = 11.8, 9.0 Hz, 2H, C_6_H_4_); 4.45 (d, *J* = 64.9 Hz, 2H, –CH_2_); 4.24–3.75 (m, 6H, –OCH); 3.74–3.32 (m, 6H, –NCH_2_); 2.26–1.81 (m, 2H, –PCH_2_); 1.63–0.77 (m, 36H, –CH_3_). ^31^P {^1^H}-NMR (20 °C, MeOD); δ ppm: 8.53. FT-IR (Nujol), cm^−1^: 616(m), 621 (m), 738 (m), 787 (m), 848 (m), 875 (m) (ν Ti–O); 955 (m), 986 (m), 1010 (vs), 1034 (m), 1083 (s) (ν P–O); 1125 (vs), 1160 (sh), 1186 (s) (ν C–O); 1260 (m) (ν P=O); 1330 (w); 1630 (w) (ν C=C).

### 3.4. Synthesis of Hybrid Nanomaterials (TiO_2_)_x_(O_3_P-R)

#### 3.4.1. Preparation of (TiO_2_)_x_(VPA) nanoparticles 

A solution based on [Ti_2_(OiPr)_6_(O_3_PC_2_H_3_)]_m_ (**1,** 0.165 mmol) + y mmol of Ti(OiPr)_4_ in THF (20 mL) was stirred for 15 min. The resulting clear solution was then injected directly using a cannula into an aqueous solution (100 mL) containing (y + 2)/10 mmol of N(nBu)_4_Br and heated until reflux. A white precipitate was immediately formed. Reflux was maintained for 3 h. The medium was then cooled to room temperature, and the white solid formed was recovered by centrifugation (4000 rpm, 1 h) and then washed once with water (75 mL) and twice with ethanol (2 × 75 mL). The resulting white solids [denoted as (TiO_2_)_x_(VPA)] were dried at 70 °C for 18 h before characterization. Values of x were been fixed in order to get a series of hybrid TiO_2_ nanomaterials with Ti/P molar ratios ranging from 2 to 200 (Table 4). For comparison, the hydrolysis of pure [Ti_2_(OiPr)_6_(O_3_PC_2_H_3_)]m (**1**) solution (y = 0) was performed to produce a (TiO_2_)_2_(VPA) sample.

#### 3.4.2. Preparation of (TiO_2_)_100_(PPA) Nanoparticles

The same procedure described below using [Ti_2_(OiPr)_6_(O_3_PC_6_H_5_)]_m_ (**2**, 0.207 mmol) and Ti(OiPr)_4_ (10.143 mmol) afforded a white powder of (TiO_2_)_100_(PPA). ^31^P-MAS NMR: 19.15, 13.95 ppm. BET (N_2_): 285 m^2·^g^−1^.

#### 3.4.3. Preparation of (TiO_2_)_100_(O_3_P-R) Nanoparticles: R = CH_2_CH_2_NHCH_2_(C_10_H_7_) (NMAPA), CH_2_CH_2_(NC_9_H_10_) (HQPA)

A modified procedure as described below (replacing THF by hot isopropanol) was used starting from precursors **3** and **4** and about 10 mmol of Ti(OiPr)_4_ to afford white powders of (TiO_2_)_100_(O_3_P-R). The hybrid nanomaterials were characterized by solid state ^31^P-MAS NMR, powder XRD, BET, elemental analysis and TGA. Powder XRD: Anatase phase (average particle size = 5 nm). (TiO_2_)_100_(NMAPA): ^31^P-MAS NMR δ ppm = 18.09; BET (N_2_): 225 m^2·^g^−1^. (TiO_2_)_100_(HQPA): ^31^P-MAS NMR δ ppm = 18.24; BET (N_2_): 322 m^2^·g^−1^.

### 3.5. PAH Batch Adsorption Test Procedure 

For all tests, a 200 ppb PAH solution was prepared by diluting in a water:ethanol (24:1) mixture 4 mL of a 500 ppm standard solution containing the US-EPA PAHs (the weight concentration of the 16 PAHs being equal in the standard stock solution). The adsorption tests were made using 10 mg of each solid material under stirring at 20 °C in 50 or 100 mL of PAH aqueous solution. Samples of 12 mL were collected after 1, 2, 4, 7, 10 and 24 h, centrifuged and 10 mL were analyzed by SPE using commercial strata-X C_18_ cartridges to extract non-adsorbed PAHs. The elution of the adsorbed compounds was carried out by passing 3 mL of ethyl acetate. The extract was concentrated at 40 °C under a stream of nitrogen to a final volume of 200 μL. The eluates were analyzed by GC-ToF in SIM mode on a DB-5MS UI column (30 m × 0.25 mm I.D, thickness 0.25 μm) with helium as carrier gas. The injection was performed in splitless mode (1 μL) at a temperature of 300 °C. The heating gradient was: 60 °C (1 min) at 320 °C (25 °C/min), 32 °C for 10 min.

## 4. Conclusions

In conclusion, a robust sol-gel process, starting from novel heteroleptic titane phosphonate-alkoxides precursors, was developed in order to tune finely the surface functionalization of nanocrystallites of titania with phosphonate ligand (PA). Series of hybrid nanomaterials bearing different amount and nature of unsaturated groups (vinyl, phenyl, naphtyl) were elaborated and thoroughly characterized. Their performances as nano-adsorbents through π−π stacking demonstrated remarkable removal efficiencies of 100% over a mixture of 16 PAHs (listed by the U.S. Environmental Protection Agency). The adsorption kinetics results have shown that systems with a ratio TiO_2_/PA = 100 reached the equilibrium after 24 h and high adsorption capacities of around 1 mg·g^−1^ per PAH were found. The selectivity of PAH sorption process depended on the nature of the unsaturated-based phosphonate ligands, on the self-agglomeration of the surface-functionalized nano-particles and on the textures of the nanomaterials. Hence, mesoporous vinyl-based titania nanomaterials have demonstrated high affinity for light PAHs while macro-, mesoporous phenyl-based one had high affinity for heavy PAHs. Such findings would be very helpful in identifying factors affecting selective PAHs remediation from aqueous media in the presence of competing species (PCB, diclofenac) or PAH mixtures.

## Figures and Tables

**Light PAH ........, Medium PAH -----, Heavy PAH ―**

**Light PAH ........, Medium PAH -----, Heavy PAH ―**

**Light PAH ........, Medium PAH -----, Heavy PAH ―**

**Light PAH ........, Medium PAH -----, Heavy PAH ―**

**Table 1:** Structures of the different phosphonic acids used in this study.

Different Phosphonic Acids	Structures		
Vinyl phosphonic acid (VPA)	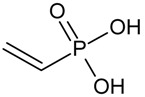
Phenyl phosphonic acid (PPA)	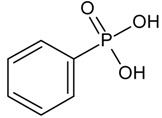
(2-{[(Naphthalen-2-yl) methyl] amino} ethyl) phosphonic acid (NMAPA)	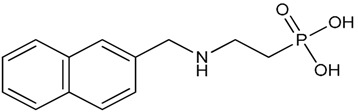
[2-(3,4-Dihydroisoquinolin-2(1*H*)-yl) ethyl]phosphonic acid (HQPA)	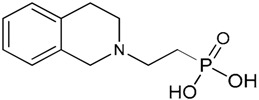

**Table 2:** Ti and P elemental analyses of the samples (TiO_2_)_x_(VPA).

Theoretical Ti/P Ratio	Experimental		
	% Ti	% P	Ti/P Ratio
25	50.69	1.03	32
50	51.29	0.72	46
75	82.00	0.82	65
100	50.03	0.35	92
125	68.43	0.36	123
150	79.84	0.35	148
200	106.31	0.36	190

**Table 3:** Ti and P elemental analysis of hybrid samples (TiO_2_)_100_(O_3_P-R).

R Group	PPA	NMAPA	HQPA
% Ti	54.52	59.62	59.06
% P	0.36	0.36	0.36
Ti/P	98	106	105

**Table 4:** Ratios of Ti/P and amounts of ***1***, [Ti_2_(OiPr)_6_(O_3_PC_2_H_3_)]_m_ and ***y***, Ti(OiPr)_4_ aimed at their co-hydrolysis

Ti/P (Molar Ratio)	2	25	50	75	100	125	150	200
**1 (mmol)**	6.711	0.165	0.165	0.165	0.165	0.165	0.165	0.165
**y (mmol)**	0.00	3.81	7.95	6.05	8.11	10.21	12.26	16.4

## Supplementary Materials

The supplementary materials are available online.
