# Supplementary material for: TiO2-Based Hybrid Nanocomposites Modified by Phosphonate Molecules as Selective PAH Adsorbents
Source: Molecules. 2018 Nov 21;23(11):3046. doi: 10.3390/molecules23113046 (PMC6278484; doi:10.3390/molecules23113046)
Supplement: Supplementary file 1 [file molecules-23-03046-s001.pdf]

Supplementary materials:

TiO_2-based hybrid nanocomposites modified by phosphonate molecules as selective PAH adsorbents

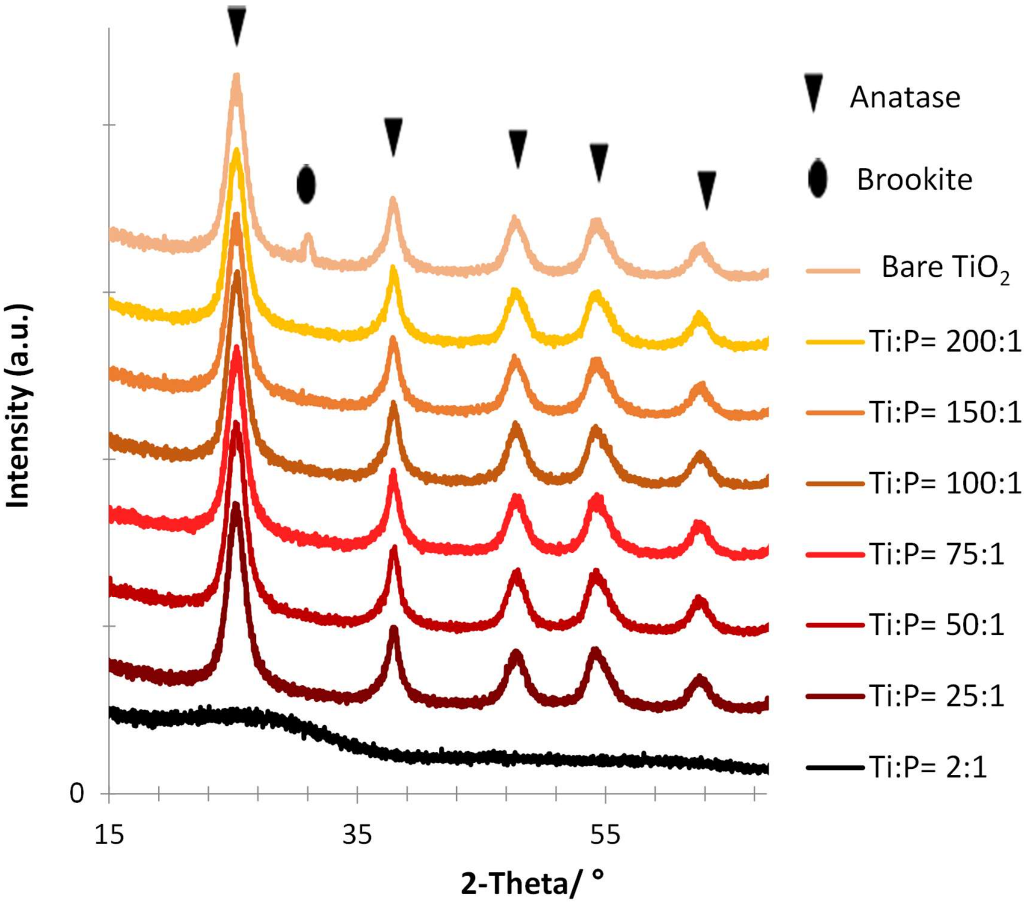

Figure S1: Powder XRD of samples $(TiO_2)_x(VPA)$ (x=2-200) and bare TiO_2

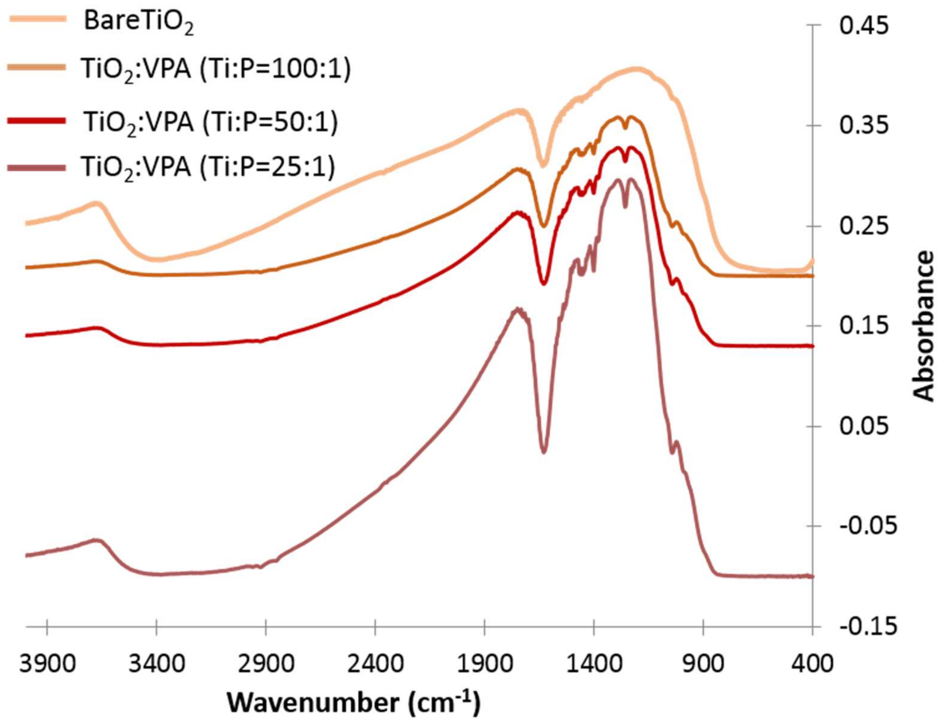

Figure S2: FT-IR spectra of samples $(TiO_2)_x(VPA)$ (x=25, 50, 100) and bare TiO_2

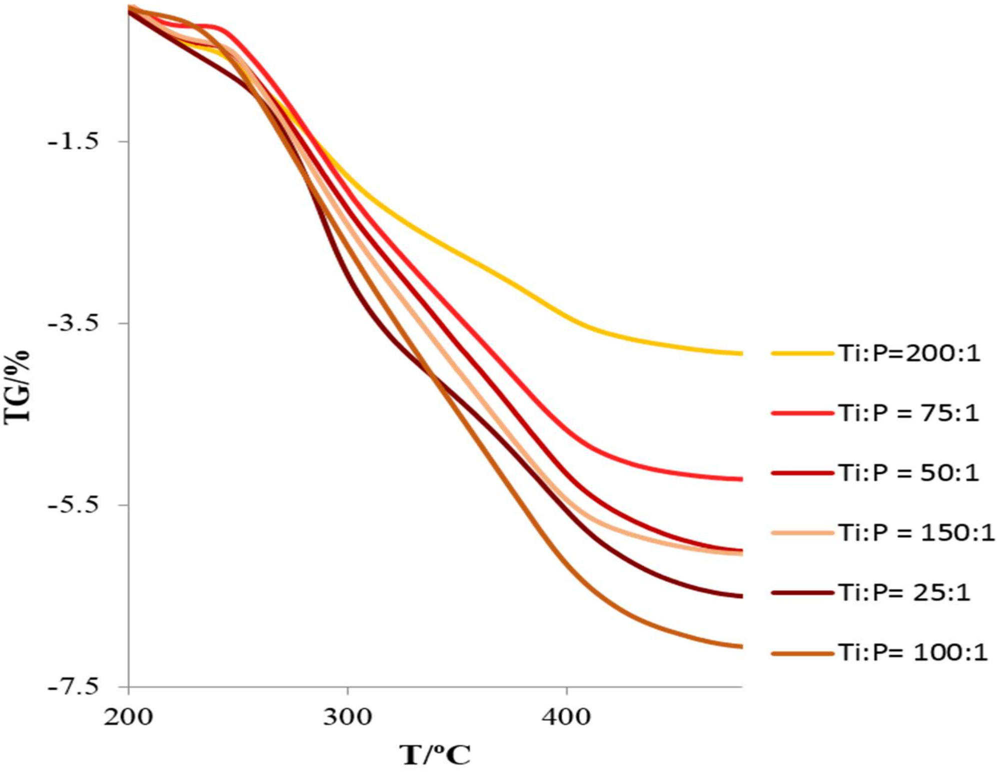

Figure S3: TGA of $(TiO_2)_x(VPA)$ samples

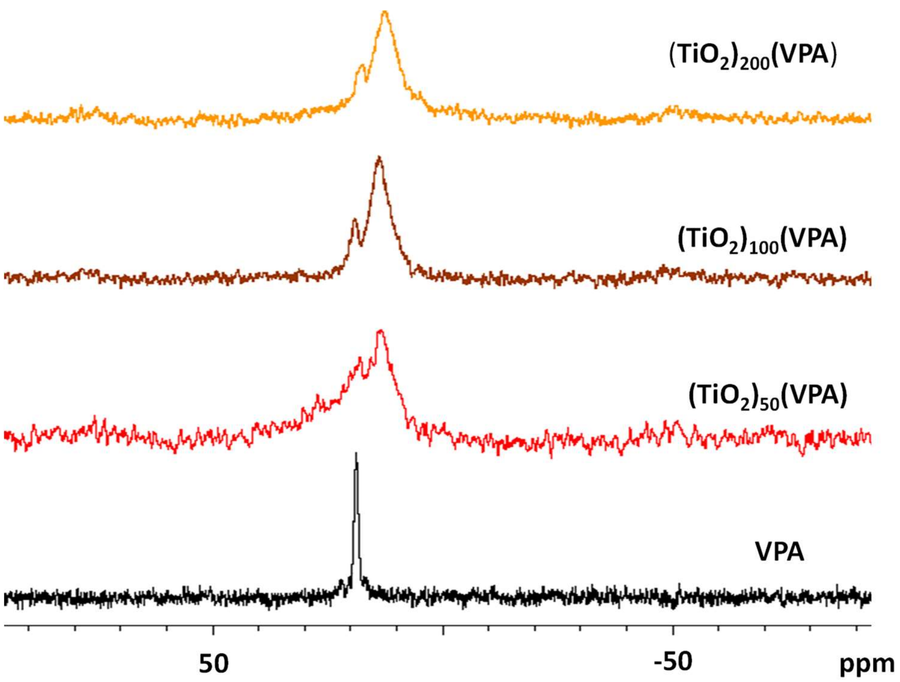

Figure S4: Solid state $^{31}P\{^1H\}$ MAS NMR spectra of VPA and hybrid samples $(TiO_2)_X(VPA)$ with x = 50, 100 et 200

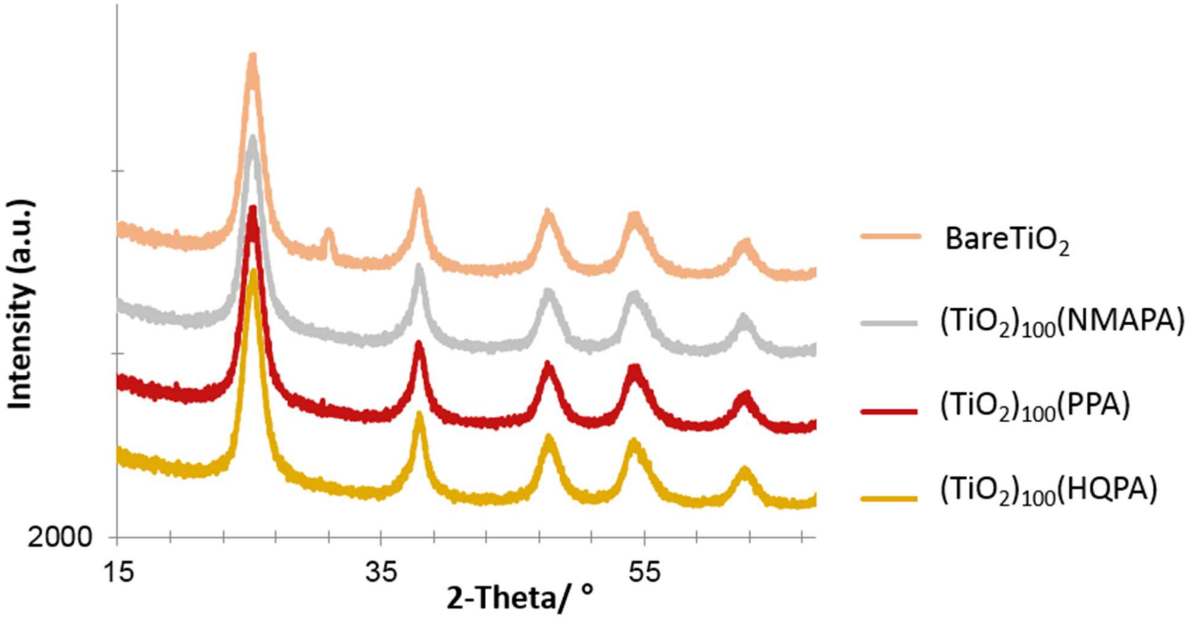

Figure S5: Powder XRD of samples $(TiO_2)_{100}(PA)$ and bare TiO_2

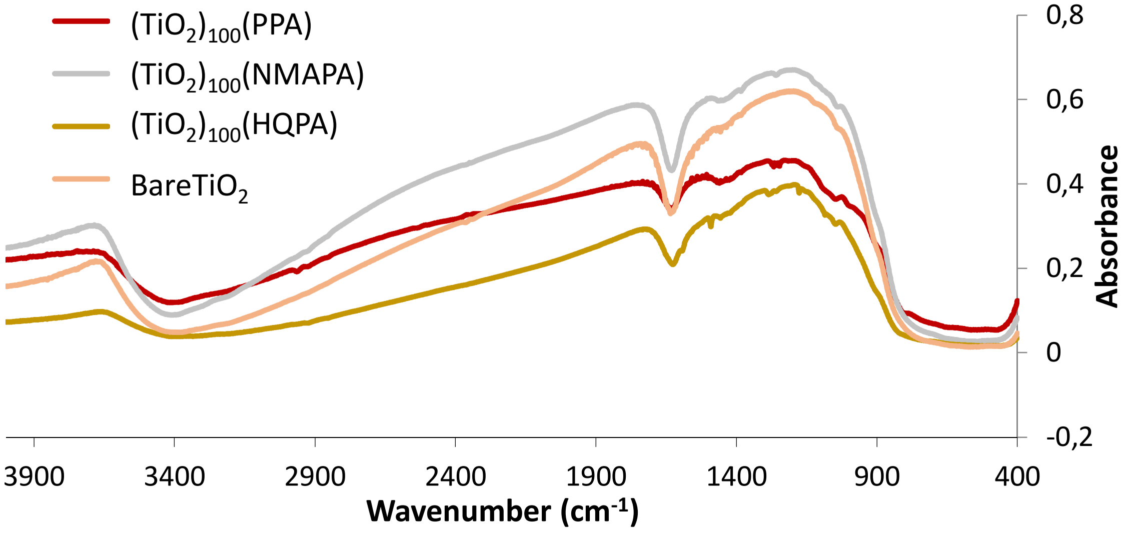

Figure S6 : FT-IR spectra of bare TiO_2 and hybrid samples $(TiO_2)_{100}$(PPA), $(TiO_2)_{100}$(NMAPA) and $(TiO_2)_{100}$(HQPA)

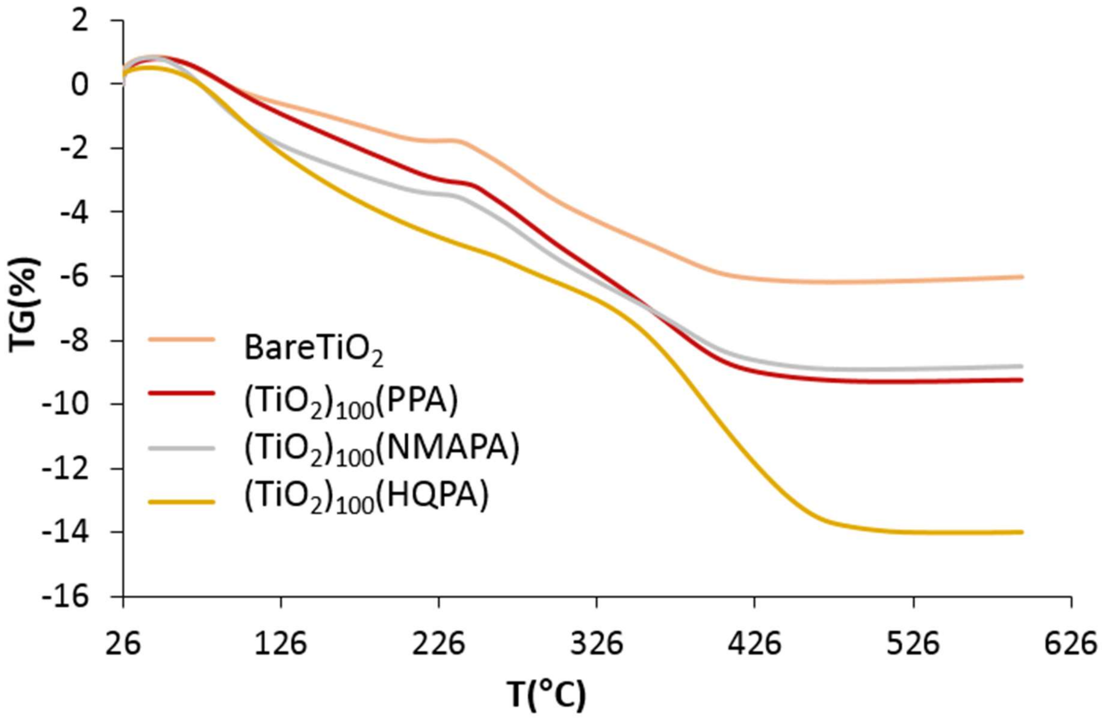

Figure S7: TGA patterns of bare TiO_2 and hybrid samples $(TiO_2)_{100}$(PPA), $(TiO_2)_{100}$(NMAPA) and $(TiO_2)_{100}$(HQPA)

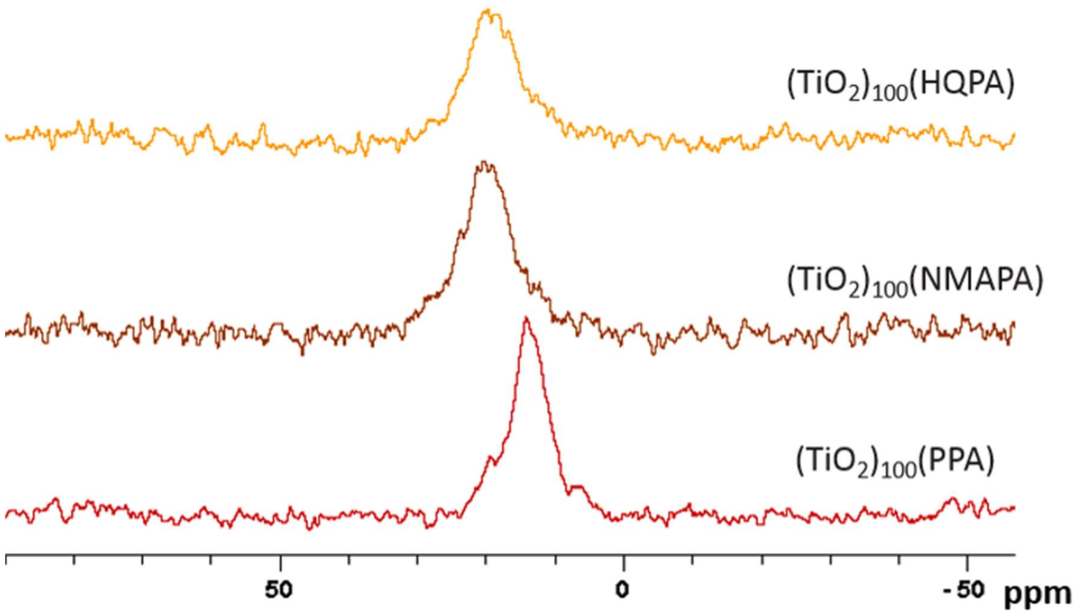

Figure S8: ^{31}P {^{1}H} MAS NMR spectra of hybrid samples $(TiO_2)_{100}$(PPA), $(TiO_2)_{100}$(NMAPA) and $(TiO_2)_{100}$(HQPA)

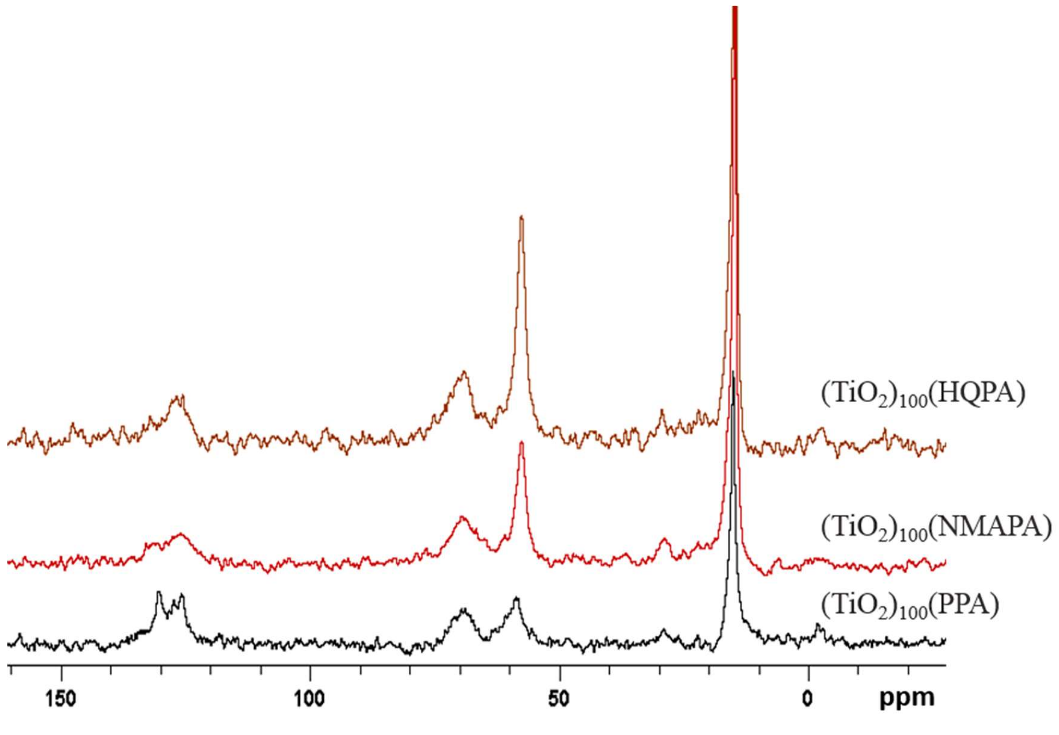

Figure S9: ^{13}C {^{1}H} MAS NMR spectra of hybrid samples $(TiO_2)_{100}$(PPA), $(TiO_2)_{100}$(NMAPA) and $(TiO_2)_{100}$(HQPA)

Table S1: Abbreviations and structures of the EPA 16 PAHs

PAH	Abbreviation	Structure
Naphtalene	NAP	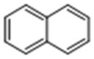
Acenaphtylene	ACY	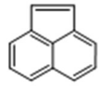
Acenaphtene	ACP	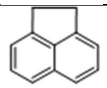
Fluorene	FLR	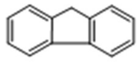
Phenanthrene	PHE	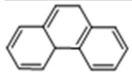
Anthracene	ANT	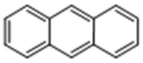
Fluoranthene	FLT	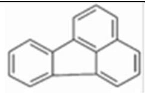
Pyrene	PYR	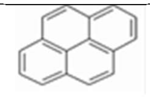
Chrysene	CHR	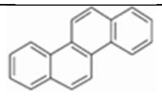
Benzo[a]Anthracene	BaA	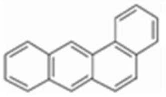
Benzo[b]fluoranthene	BbF	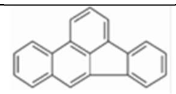
Benzo[k]fluoranthene	BkF	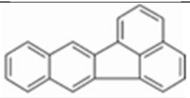
Benzo[a]pyrene	BaP	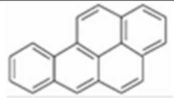
Indeno[1.2.3-cd] pyrene	IcP	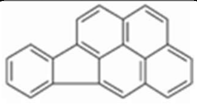
Dibenz[a,h]anthracene	DbA	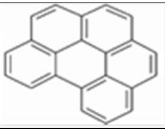
Benzo[ghi]perylene	BgP	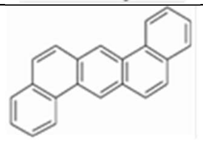

Table S2: BET surface and porous volume of bare TiO_2 and hybrid $(TiO_2)_{100}(O_3P\text{-}R)$ nanomaterials

**Material**	**Porous volume (cm³ / g)**	**BET surface area (m² / g)**
TiO_2	0.49	250
$(TiO_2)_{100}$(VPA)	0.39	269
$(TiO_2)_{100}$(PPA)	0.50	285
$(TiO_2)_{100}$(NMAPA)	0.51	225
$(TiO_2)_{100}$(HQPA)	0.50	322

Table S3: Summary of kinetic parameters adsorption of $(TiO_2)_{100}$(VPA) toward Benzo(a)pyrene

**Model**	**Parameters**
Pseudo-first-order	
k_1 (min^{-1})	0.00207
q_e ($mg\ g^{-1}$)	0.00855
R^2	0.1403
Pseudo-second-order	
k_2 ($g\ mg^{-1}\ min^{-1}$)	1.383
q_e ($mg\ g^{-1}$)	0.850
R^2	1.000
